# A Pathophysiological Model of Non-Alcoholic Fatty Liver Disease Using Precision-Cut Liver Slices

**DOI:** 10.3390/nu11030507

**Published:** 2019-02-27

**Authors:** Grietje H. Prins, Theerut Luangmonkong, Dorenda Oosterhuis, Henricus A. M. Mutsaers, Frank J. Dekker, Peter Olinga

**Affiliations:** 1 Department of Pharmaceutical Technology and Biopharmacy, University of Groningen, 9712VM Groningen, The Netherlands; G.G.H.Prins@rug.nl (G.H.P.); theerut.lua@mahidol.edu (T.L.); D.Oosterhuis@rug.nl (D.O.); H.A.M.Mutsaers@rug.nl (H.A.M.M.); 2 Department of Pharmacology, Faculty of Pharmacy, Mahidol University, 10400 Bangkok, Thailand; 3 Department of Chemical and Pharmaceutical Biology, University of Groningen, 9712VM Groningen, The Netherlands; F.J.Dekker@rug.nl

**Keywords:** NAFLD, non-alcoholic fatty liver disease, ex vivo, pathophysiological model, metabolism, steatosis

## Abstract

Non-alcoholic fatty liver disease (NAFLD) is a common liver disorder closely related to metabolic syndrome. NAFLD can progress to an inflammatory state called non-alcoholic steatohepatitis (NASH), which may result in the development of fibrosis and hepatocellular carcinoma. To develop therapeutic strategies against NAFLD, a better understanding of the molecular mechanism is needed. Current in vitro NAFLD models fail to capture the essential interactions between liver cell types and often do not reflect the pathophysiological status of patients. To overcome limitations of commonly used in vitro and in vivo models, precision-cut liver slices (PCLSs) were used in this study. PCLSs, prepared from liver tissue obtained from male Wistar rats, were cultured in supraphysiological concentrations of glucose, fructose, insulin, and palmitic acid to mimic metabolic syndrome. Accumulation of lipid droplets was visible and measurable after 24 h in PCLSs incubated with glucose, fructose, and insulin, both in the presence and absence of palmitic acid. Upregulation of acetyl-CoA carboxylase 1 and 2, and of sterol responsive element binding protein 1c, suggests increased de novo lipogenesis in PCLSs cultured under these conditions. Additionally, carnitine palmitoyltransferase 1 expression was reduced, which indicates impaired fatty acid transport and disrupted mitochondrial β-oxidation. Thus, steatosis was successfully induced in PCLSs with modified culture medium. This novel ex vivo NAFLD model could be used to investigate the multicellular and molecular mechanisms that drive NAFLD development and progression, and to study potential anti-steatotic drugs.

## 1. Introduction

Non-alcoholic fatty liver disease (NAFLD), also known as hepatic steatosis, is a condition in which there is an abnormally high lipid deposition in the liver that is unrelated to excessive alcohol intake [1,2]. The severity of NAFLD varies, and an estimated 20% of NAFLD patients develop non-alcoholic steatohepatitis (NASH), [3] which can progress to liver cirrhosis or hepatocellular carcinoma [1,2,3,4]. NAFLD is strongly associated with metabolic syndrome, in particular the metabolic abnormalities hyperinsulinemia and hyperglycemia [4,5,6,7,8,9].

The supraphysiological concentrations of insulin and glucose associated with these pathological conditions induce lipogenic genes through the action of sterol regulatory element binding protein-1c (SREBP-1c), and carbohydrate responsive element binding protein (MLXIPL), respectively. SREB-1c and MLXIPL induce acetyl-CoA carboxylase 1 (ACC1), which is involved in the synthesis of malonyl-CoA—a substrate for fatty acid synthesis and inhibitor of fatty acid oxidation. This chain of reactions results in a misbalance of carbohydrate and lipid metabolism [10,11,12], and instead of oxidizing lipids, triglyceride esterification and thus de novo lipogenesis (DNL) is increased [13]. For example, diabetes mellitus is a major co-morbid disease in NAFLD patients [10,11].

Glucose is not the only saccharide that can induce de novo lipogenesis. Fructose has the same basic properties [14,15,16,17], but a big difference is that fructose is mainly metabolized in the liver, whereas glucose can be metabolized throughout the whole body [16,18]. Therefore, the negative effects of fructose consumption, which has increased dramatically over the years, will mostly affect the liver [19].

While de novo lipogenesis is a key source of triglycerides, another source are the lipids that are present in the liver. Hyperlipidemia, an elevated amount of blood lipids, and especially hypertriglyceridemia, leads to an increased hepatic uptake of lipids and thereby contributes to hepatic steatosis [7,20].

Besides increased lipid uptake and lipid synthesis, reduced lipolysis and secretion also play an important role in the accumulation of fat. Under normal circumstances, long-chain fatty acids are transported from the cytosol to the mitochondrial matrix where they undergo β-oxidation [21]. Transport to the mitochondrial matrix depends on carnitine palmitoyltransferase 1 (CPT1) and carnitine palmitoyltransferase 2 (CPT2) [22]. 

Additionally, lipids are directly involved in lipotoxicity [21,23]. The lipotoxicity has been extensively investigated in vitro using palmitic acid, which is the most abundant saturated fatty acid in the human body [24,25,26]. While the molecular mechanism behind palmitate’s ability to induce lipotoxic reactive oxygen species is not entirely clear, it is assumed that fatty acid oxidation plays an important role [27]. Lipotoxicity and disruption of mitochondrial β-oxidation are considered the most harmful factors in NAFLD development and progression [26].

NAFLD can only partly be managed by modifications in diet and lifestyle [28,29]. To date, the mechanisms underlying NAFLD development and the progression to NASH remain elusive. Consequently, there are no approved pharmacological interventions for NAFLD or NASH. 

Several in vitro models are used to study the pathogenesis of NAFLD [30,31], but these do not accurately reflect the pathophysiological status of NAFLD patients since many models do not recapitulate the multicellular nature of the disease [11,32,33]. In the pathogenesis of NAFLD and related liver diseases, communication between hepatocytes, Kupffer cells, and hepatic stellate cells is essential [32,33]. The most prominent advantage of precision-cut liver slices (PCLSs) is the preserved multicellular environment allowing for the interplay between various liver cell types [31,34]. PCLSs have previously been shown to be a useful model to study multicellular diseases [35,36], and may be a useful model to study the pathophysiological processes in NAFLD. Therefore, the aim of this study was to induce liver steatosis in PCLSs, by using culture conditions that reflect metabolic syndrome. 

## 2. Methods

### 2.1. Animals

Male Wistar rats, aged 12 to 16 weeks, were purchased from Charles River (Sulzfeld, Germany). Experiments were approved by the Animal Ethical Committee of the University of Groningen. 

### 2.2. Precision-Cut Liver Slices (PCLSs)

PCLSs, with an estimated thickness of 250–300 μm, were prepared using a Krumdieck Tissue Slicer (Alabama Research and Development, Munford, AL, USA) [37], and cultured under continuous supply of 80% O_2_ and 5% CO_2_, as previously described [36]. PCLSs were cultured up to 48 h and culture media was refreshed every 24 h. 

### 2.3. Culture Media

Williams medium E with Glutamax (Invitrogen, Bleiswijk, the Netherlands), supplemented with gentamycin (50 mg/mL; Invitrogen), was used as control medium. To mimic metabolic syndrome, supraphysiological concentrations of glucose (Merck, Darmstadt, Germany), fructose (Merck, Darmstadt, Germany), human insulin (Sigma-Aldrich, St. Louis, MO, USA), and palmitic acid (Sigma-Aldrich, St. Louis, MO, USA) were added to the medium. The experimental concentrations (Table 1) were based on human serum concentrations [5,6,7,11,32,33] and in vivo rodent portal vein concentrations [38,39,40]. 

Palmitic acid was solubilized using bovine serum albumin (BSA; Sigma-Aldrich, St. Louis, MO, USA). Palmitic acid was briefly dissolved in 0.1 M sodium hydroxide (Merck, Darmstadt, Germany) at 70 °C, and then mixed with preheated BSA solution at 55 °C. The same concentration of BSA (0.04%), without palmitic acid, was added to media not containing palmitic acid. This concentration of BSA had no effect on PCLS viability. 

### 2.4. Oil Red O Staining 

Cryosections were prepared by embedding fresh PCLSs in KP-cryocompound (Klinipath, Duiven, the Netherlands) on dry ice. Tissue sections, 4 μm in thickness, were prepared perpendicular to the surface of PCLSs. Steatosis was evaluated by staining lipid droplets with Oil Red O. Cryosections were fixed with 4% formaldehyde/PBS for 10 min before staining with Oil Red O solution (0.6% Oil Red O in 36% 2-propanol) for 10 min at room temperature. Sections were counterstained with haematoxylin and fixed with Aquatex (Merck, Darmstadt, Germany). Pictures were taken with a BX41 microscope (Olympus America Inc, Center Valley, PA, USA, 400× magnification), and analyzed using ImageScope software (v12.3.2.8013, Aperio, Vista, CA, USA). A ratio of red pixels to blue pixels (lipid droplets per nucleus) was used to examine the change in fat content. Three PCLSs from each treatment were used per experiment. 

### 2.5. ATP Determination

Viability was assessed by determining ATP content of the slices using a bioluminescence kit (Roche Diagnostics, Mannheim, Germany) as previously described [37]. Values for each group were expressed as relative percentage of the 24 h control. 

### 2.6. Protein Estimation

Total protein content of the slices was estimated using a Lowry assay (BioRad DC Protein Assay, Hercules, CA, USA). Values were expressed as change in protein content as compared to the 24 h control. 

### 2.7. Triglyceride Quantitation

PCLSs were snap-frozen and stored at −80 °C. Subsequently, the slices were homogenized in Tris-buffered saline. Fat was isolated using the Bligh & Dyer method [41]. The amount of triglycerides was determined using a Trig/GB kit (Roche Molecular Biochemicals, Almere, the Netherlands) according to the protocol provided by the manufacturer. Values were calculated using the absorption at 540 nm after one hour, and are displayed as the change in percentage as compared to the control (CTR). 

### 2.8. Quantitative Real-Time PCR 

Expression of key genes in carbohydrate and lipid metabolism was assessed using quantitative real-time PCR. For each experiment, three slices were pooled per condition, snap-frozen in liquid nitrogen, and stored at −80 °C. RNA was isolated using the RNeasy Lipid Tissue Mini Kit (Qiagen, Venlo, the Netherlands). The Reverse Transcription System (Promega, Leiden, the Netherlands) was used to reverse transcribe RNA. Expression of genes related to metabolism, inflammation, and ER stress was determined using TaqMan primers and probes and SYBR Green primers, the sequences of which can be found in Table S1, Supplementary Materials (ThermoFisher Scientific, Waltham, MA, USA). Quantitative real-time PCR was performed using a VIIA7 thermal cycling system (Applied Biosystems, Carlsbad, CA, USA). For TaqMan primers and probes, the 1X FastStart Universal Probe Master (Roche, Almere, the Netherlands) was used. The thermal cycling conditions were one cycle at 95 °C for 10 min, followed by 45 cycles starting with 95 °C for 15 s, then 60 °C for 30 s, and ending with 72 °C for 30 s. For SYBR Green primers, the FastStart Universal Sybr Green Master (Roche, Almere, The Netherlands) was used. On the VIIA7 thermal cycling system, the process started with a 10 min hold at 95 °C, followed by 40 cycles that consisted of 15 s at 95 °C, 30 s at 60 °C, and 30 s at 72 °C. Ct values were corrected for the Ct values of *Ywhaz* (ΔCt) and compared to control (ΔΔCt). Results are displayed as fold induction (2^−ΔΔCt^).

### 2.9. Statistics

For each liver, three PCLSs were used per condition. Each experiment was performed at least three times. Results are expressed as means ± standard error of the mean (SEM) and compared to the control group, using a one-way ANOVA with Dunnett’s post hoc analysis, unless specified otherwise. Results were considered statistically significant when the calculated *p*-value was smaller than 0.05. 

## 3. Results

### 3.1. PCLS Characteristics

**Figure 1:**
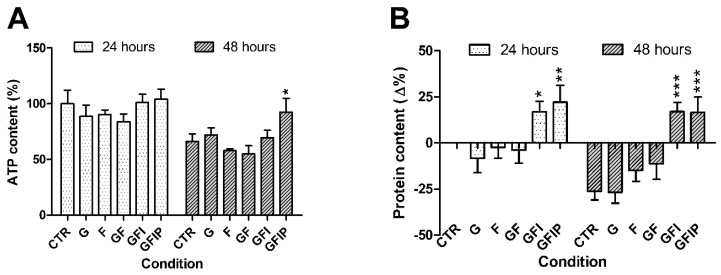
(**A**) ATP content of precision-cut liver slices (PCLSs) after 24 and 48 h. Data is presented as mean percentage of 24 h control ± SEM (*n* = 5). Using a one-way ANOVA, all conditions were compared to the relative control. * *p* < 0.05 (**B**) Difference in protein content after 24 and 48 h. Data is expressed as mean difference as compared to the 24 h control ± SEM (*N* = 5). Significance was determined using a one-way ANOVA, comparing all conditions from a time point to the control of that time point. * *p* < 0.05, ** *p* < 0.01, *** *p* < 0.001. CTR = control, G = Glucose, F = Fructose, GF = Glucose and fructose, GFI = Glucose, fructose, and insulin, GFIP = Glucose, fructose, insulin, and palmitic acid.

PCLSs remained viable, as assessed by ATP content, in all conditions and at all time points (Figure 1A). In control PCLSs, ATP content was lowered from 100% ± 11% at 24 h of incubation to 66% ± 6% at 48 h. This difference was not statistically significant.

Fructose-containing media (F and GF) seemed to reduce ATP content in PCLSs, especially after 48 h. However, this reduction was not significant. After 48 h, ATP content of slices incubated in medium GFI was not different from the 48 h control, but addition of palmitic acid resulted in a significantly increased ATP content. 

Figure 1B depicts the changes in protein content of PCLSs, as compared to the 24 h control. Measured protein levels were higher in PCLSs cultured in the insulin-containing media (GFI and GFIP) after 24 h, as compared to the 24 h CTR. Protein content of other conditions did not differ from control. The amount of protein in PCLSs was significantly reduced over time, except for PCLSs in GFI and GFIP media, which retained increased protein levels after 48 h. 

**Figure 2:**
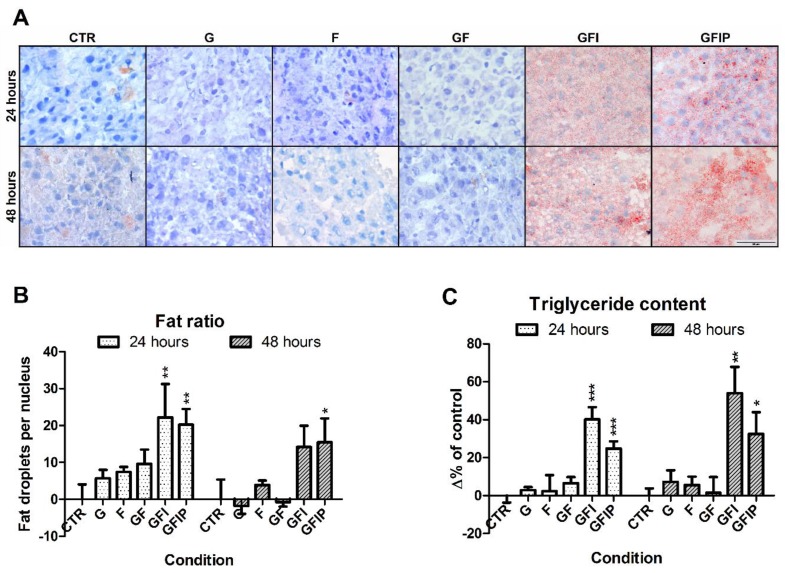
(**A**) Representative Oil Red O stained PCLS sections, cultured in different culture media, for 24 h and 48 h. CTR = control, G = Glucose, F = Fructose, GF = Glucose and fructose, GFI = Glucose, fructose, and insulin, GFIP = Glucose, fructose, insulin, and palmitic acid. (**B**) Fat-to-nucleus ratio. Oil Red O stained sections were used to determine the ratio of fat droplets per nucleus. Data is shown as the mean change in percentage of the ratio ± SEM (*N* = 4). (**C**) Difference in measured triglyceride content after 24 h and 48 h. Data is expressed as mean difference in percentages of the 24 h untreated control ± SEM (*n* = 5–6). Significance was determined using a one-way ANOVA, comparing all conditions from a time point to the control of that time point. * = *p*-value < 0.05, ** = *p*-value < 0.01, *** = *p*-value < 0.001.

Morphology of PCLSs after 24 and 48 h of incubation in the different media compositions is shown in Figure 2. There are no clear signs of cellular damage in the form of pyknosis and necrosis after 24 or 48 h. 

### 3.2. Hepatic Steatosis in PCLSs

Steatosis was evaluated microscopically in PCLSs using an Oil Red O staining. As shown in Figure 2A, few lipid-rich droplets were visible after 24 h of incubation in media that contained glucose (G), fructose (F), and glucose and fructose (GF). In contrast, an abundance of small-size lipid droplets was seen in PCLSs cultured in media containing glucose, fructose, and insulin (GFI) as well as in glucose, fructose, insulin, and palmitic acid (GFIP). After 48 h, the pattern of steatosis was similar. No lipid droplets were observed in PCLS cultured in media that contained glucose, fructose and palmitic acid (Supplementary Figure S1). 

In order to confirm the microscopic evaluation, liver steatosis was quantified using a fat-to-nucleus ratio calculation. From the 24 h- to the 48 h-time point, the fat-to-nucleus ratio did not increase in control PCLSs (Figure S2, Supplementary Materials). Figure 2B shows that when compared to control, culturing in medium GFI and in medium GFIP for 24 h resulted in a 20% increase of fat per nucleus. After 48 h, in comparison to the 48 h control, the amount of fat droplets per nucleus increased 15% for both GFI and GFIP. 

Figure 2C shows the level of triglycerides that were measured in PCLSs, as a difference in percentage of the 24 h control. For conditions G, F, and GF, no difference in the content of triglycerides could be determined at either time point when comparing to control PCLSs. After 24 h and 48 h, the triglyceride content was significantly increased in PCLSs cultured in medium containing saccharides and insulin, both in the absence and presence of palmitic acid (34% and 27%, respectively, after 24 h, and 52% and 38% after 48 h). 

### 3.3. Lipid Metabolism

**Figure 3:**
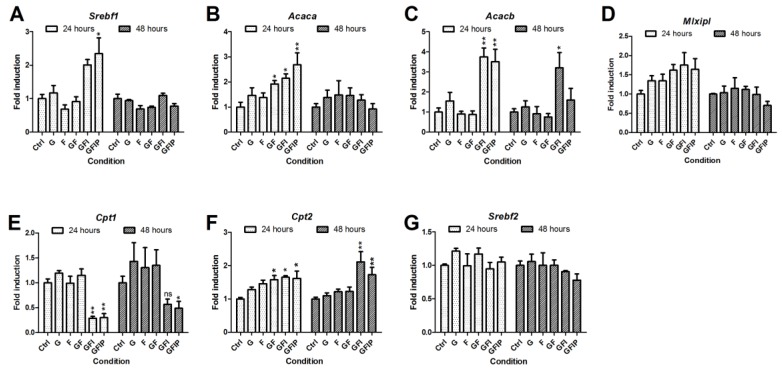
Relative mRNA expression of genes related to lipid metabolism, specifically (**A**) *Srebf1*, (**B**) *Acaca*, (**C**) *Acacb*, (**D**) *Mlxipl*, (**E**) *Cpt1*, (**F**) *Cpt2*, and (**G**) *Srebf2*. Values are displayed as mean fold induction ± SEM (*n* = 3). Significance was determined using a one-way ANOVA comparing conditions to the 24 h or 48 h control, respectively. * = *p*-value < 0.05, ** = *p*-value < 0.01, ns = *p*-value > 0.05. G = Glucose, F = Fructose, GF = Glucose and fructose, GFI = Glucose, fructose, and insulin, GFIP = Glucose, fructose, insulin, and palmitic acid.

To investigate whether the accumulation of lipid droplets (Figure 2) was a consequence of culture medium modifications, metabolic processes were further investigated by determining gene-expression genes related to lipid metabolism. Culturing PCLSs in different media had no significant effects on the RNA yield (data not shown). Figure 3 shows the relative fold induction of genes involved in lipid metabolism, as compared to the 24 h and 48 h control, respectively. Sterol regulatory element binding protein 1c (*Srebf1*) expression was only increased after 24 h in PCLSs cultured in GFI and GFIP medium, as seen in Figure 3A. An increased acetyl-CoA carboxylase 1 (*Acaca*) and acetyl-CoA carboxylase 2 (*Acacb*) fold induction is seen in Figure 3B,C. This increase was significant for the groups GFI and GFIP after 24 h. The effect on *Acaca* was no longer visible after 48 h, but the *Acacb* fold induction remained higher. Over time, *Acaca* expression remained constant and *Acacb* gene expression increased 3.8-fold in control PCLSs, as is seen in Figure S3, Supplementary Materials. The first 24 h carbohydrate responsive element binding protein (*Mlxipl*) fold induction seemed to be upregulated by the different culture media, however, not significantly. After 48 h, no upregulation was observed (Figure 3D).

Figure 3E,F shows that carnitine palmitoyltransferase 1 (*Cpt1*) expression is lower for PCLSs cultured in GFI and GFIP medium, both after 24 and 48 h. This in contrast to carnitine palmitoyltransferase 2 (*Cpt2*) expression, which was significantly increased under the same conditions. 

None of the media influenced sterol regulatory element binding protein 2 (*Srebf2*) expression (Figure 3G). 

### 3.4. Inflammation, Endoplasmic Reticulum Stress, and Fibrosis in PCLSs

**Figure 4:**
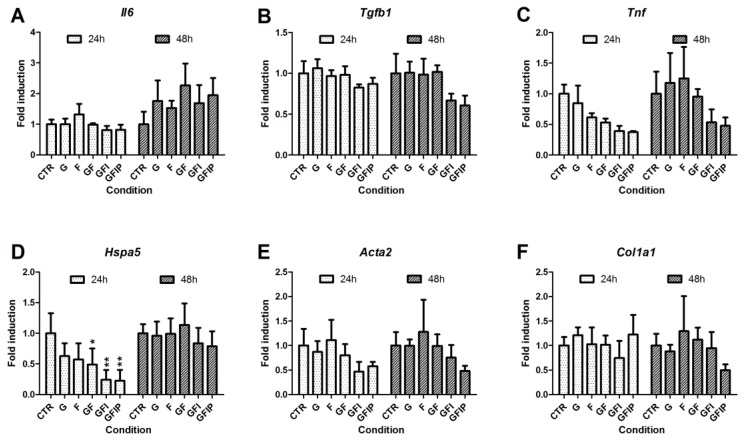
Expression of mRNA related to inflammation, endoplasmic reticulum stress, and fibrosis. Expression of genes responsible for inflammation, (**A**) *Il6*, (**B**) *Tgfb1*, and (**C**) *Tnf*, for endoplasmic reticulum stress, (**D**) *Hspa5*, and for fibrosis, (**E**) *Col1a1* and (**F**) *Acta2*, is expressed as mean relative fold induction ± SEM (*n* = 3). A one-way ANOVA comparing conditions to their respective control was used to determine significance. CTR = control, G = Glucose, F = Fructose, GF = Glucose and fructose, GFI = Glucose, fructose and insulin, GFIP = glucose, fructose, insulin, and palmitic acid. * = *p*-value < 0.05, ** = *p*-value < 0.01.

To investigate the effects of the different culture media on the inflammatory responses of PCLSs, the expression of interleukin-6 (*Il6*), transforming growth factor beta (*Tgfb*), and tumor necrosis factor-alpha (*Tnf*) was determined (Figure 4). Over time, *Il6* and *Tnf* expression increased in PCLSs. *Tgfb* expression remained constant. While no difference could be seen in *Il6* expression between slices cultured in different media, both *Tgfb* and *Tnf* expression seemed to be reduced in the conditions GFI and GFIP. These changes were not significant.

Heat shock protein family A member 5 (*Hspa5*), more commonly referred to as GRP78/BiP, expression was measured to get an indication of endoplasmic reticulum (ER) stress caused by the different media. The expression of *Hspa5* increased significantly over time in control PCLSs, as depicted in Figure S4, Supplementary Materials. Figure 4D shows a significant reduction of *Hspa5* expression after 24 h incubation in media GF, GFI, and GFIP as compared to CTR. After 48 h, no differences were seen.

To see whether the fat accumulation due to the studied media could already induce a fibrotic response in PCLSs, the expression of actin, alpha 2, smooth muscle, aorta (*Acta2*) and collagen type I alpha 1 chain (*Col1a1*) was examined. No different gene expression was observed for either gene in PCLSs cultured in different media, as is shown in Figure 4E,F. 

## 4. Discussion

The burden of NAFLD for patients and society is high, and the mechanism of NAFLD development remains elusive. One of the problems is the lack of good in vitro models to study this disease, as many models do not contain the different cell types that drive pathogenesis [32,33]. In PCLSs, the different cell types are present in their physiological environment [31,34], which has been beneficial in studies on drug metabolism, drug toxicity, and fibrosis [35,36,37,42]. In this study, steatosis was successfully induced in PCLSs derived from rat liver tissue. 

The biggest risk factors for NAFLD are those related to metabolic syndrome, in particular hyperglycemia, dyslipidemia, and hyperinsulinemia [5,6,7,10,11,12]. Therefore, glucose, fructose, insulin, and palmitic acid were selected as supplements for the NAFLD–PCLS culture. As the absolute pathophysiological portal concentration range of these supplements in NAFLD patients is unknown, supraphysiological concentrations based on pathophysiological serum concentrations in NAFLD patients [11,32,33], and previously measured rodent portal concentrations [38,39,40], were used.

Morphological evaluation and ATP content show that PCLSs remain viable in every condition. After 48 h, only a small reduction in ATP content was seen for PCLSs that were cultured in fructose-containing media. This reduction could be explained by active fructose phosphorylation, which has no feedback loop and only ends when either fructose or ATP is depleted [43]. As no reduction of RNA or protein content was observed, the reduction of ATP did not seem to be a problem for PCLSs. The reduction in total protein content over time is a normal phenomenon in PCLSs [44]. The increased protein content of PCLSs cultured in media that contain insulin suggests that insulin promotes protein synthesis and possibly reduces protein catabolism, which is in line with other studies [45,46]. Additionally, protein synthesis could be increased due to the endoplasmic reticulum (ER) stress present in PCLSs [47].

In contrast to other in vitro studies, palmitic acid did not impact PLCS viability. The main toxic effect of palmitic acid in obese and insulin-resistant individuals is impaired ATP synthesis [27]. This toxicity has been observed in vitro as well, and is caused by palmitate-induced mitochondrial dysfunction [24,25,26]. There are several possible explanations for the absence of lipotoxicity in our model. First, the antioxidant glutathione is lacking in many in vitro models, but present in PCLSs [48,49]. Glutathione in PCLSs might be able to counteract palmitic acid-induced reactive oxygen species. Additionally, the concentration of palmitic acid in this model might have been too low to cause mitochondrial dysfunction, and was used for cellular energy production (as was found after 48 h in the slices) [27]. 

The amount of steatosis that resulted from culturing in different media was determined by means of Oil Red O staining and triglyceride measurement. In the absence of insulin, no steatosis occurs in PCLSs regardless of other medium components. This indicates that exogenous insulin promotes the formation and accumulation of lipid droplets in this pathophysiological PCLS model, which is in line with other studies [50]. This could be caused by insulin-dependent de novo lipogenesis and *Srebf1* processing [50]. The unaltered *Srebf2* gene expression in the PCLSs indicates that there was no additional cholesterol formation in PCLSs and that lipid droplets therefore most likely consisted of triglycerides [11].

The lipid droplets that accumulated in PCLSs in this study can be characterized as microvesicular steatosis. This is in line with other in vitro NAFLD studies [51,52,53], but differs from the macrovesicular steatosis found in various in vivo models of NAFLD [30]. A possible explanation for the size difference in lipid droplets between the models is the difference in the duration of steatosis induction. For in vivo studies, this timeframe comprises months, while in vitro studies are generally limited to several days. In shorter in vivo studies, microvesicular lipid droplets have been observed [54].

In this early onset of steatosis, another factor is the different manner of lipid droplet formation, which may be caused by either reduced mitochondrial β-oxidation or de novo lipogenesis [21].

In this study, gene expression of *Srebf1* and *Mlxipl* and gene expression of their target genes *Acaca* and *Acacb* [11] were used as a measure of de novo lipogenesis. All the aforementioned genes were upregulated in PCLSs where onset of steatosis was observed. While protein expression for these genes was not determined, it has been shown that increased mRNA expression of de novo lipogenesis genes was related to increased protein expression of these genes [55,56,57].

Previous studies have shown that impaired mitochondrial β-oxidation might be the cause of microvesicular steatosis in vivo [10,58]. To find out whether mitochondrial β-oxidation plays a role in steatosis in PCLSs, *Cpt1* mRNA expression was examined. CPT1 transports fatty acids from the cytosol to mitochondria, and thereby catalyzes the rate-limiting step of fatty acid oxidation [11]. CPT1 is inhibited by the substrate that the acetyl-CoA carboxylases (ACC) produce. In line with increased *Acc* gene expression, a reduction in *Cpt1* gene expression was found. This phenomenon has been observed in in vivo and in vitro studies [10,11]. *Cpt2* expression was not downregulated as CPT2 is not inhibited by the substrate produced by ACC. Increased CPT2 levels have previously been linked to stress, which could also be the case in PCLSs [59]. 

These results indicate that both increased lipid synthesis and reduced lipid breakdown are connected to the steatosis in this PCLS model, which is in line with in vivo studies [22]. Furthermore, the results support the idea that acetyl-CoA 1 and 2 inhibition might be an interesting target to halt de novo lipogenesis [60].

In this study, changes in the expression of *Hspa5*, also known as glucose-regulated protein, 78kDa/binding-immunoglobulin protein (GRP78/BiP), were observed. HSPA5 is the master regulator of endoplasmic reticulum (ER) homeostasis and interacts with the three major unfolded protein response (UPR) branches, which have direct effects on lipid synthesis [61,62]. Over time, expression of *Hspa5* increased in control PCLSs. HSPA5 gene expression is linked to HSPA5 protein expression [63], and is induced in response to ER stress [62]; these results indicate that PCLS preparation or culturing leads to ER stress. Increased HSPA5 expression has been shown to protect against hepatic steatosis, whereas HSPA5 loss induces it [62,64]. Additionally, loss of HSPA5 induces phosphorylation of c-Jun N-terminal kinases (JNK), which has been linked to NASH [65]. Upon measuring *Hspa5* in PCLSs, a significant reduction was found when culturing in medium containing sugars, insulin, and fat. While the exact relationship between UPR signaling and lipogenesis is unknown [66], the decrease might be linked to increased *Acaca* expression [65] and increased *Srebf1* expression [67], both of which were also observed in PCLSs. As the HSPA5 decrease in PCLSs is in line with observations in NAFLD/NASH patients [68,69], it might be interesting to study the relation between ER stress, UPR, and fatty liver disease in this PCLS model.

NAFLD has different stages, the first being fatty liver. This stage is characterized by a certain amount of steatosis and an absence of inflammation or fibrosis. A percentage of patients in this first stage will develop the more harmful condition non-alcoholic steatohepatitis, which is characterized by inflammation of fatty liver tissue. In this study, steatosis was observed but no inflammation or fibrosis was measured on a gene level. Therefore, one of the next steps in NAFLD–PCLS is to induce inflammation. Since it is possible in this PCLS model to control which factors are present and in which order, it could be used to gain more insight into NAFLD development and progression. For example, the model could be used to clarify whether there is a specific order in which inflammation and steatosis drive NAFLD/NASH progression, or whether they work side by side [5,23].

## 5. Conclusions

In conclusion, microvesicular steatosis was induced in precision-cut liver slices by mimicking the NAFLD risk factors. Gene expression data suggest that early steatosis was achieved through both de novo lipogenesis and reduced mitochondrial β-oxidation. While gene expression indicated the presence of ER stress, the culture media did not have a negative effect on PCLS viability, or induce an inflammatory or fibrotic response. More studies are needed to determine whether a proper pathophysiological NAFLD–PCLS model was developed, and the limitations of this PCLS model, such as the short incubation time and the absence of inter-tissue communication, should be taken into account. Precision-cut liver slices derived from human tissue would even better enable extrapolation to the situation in humans. This PCLS model may be pivotal in investigating the multicellular molecular mechanisms behind NAFLD development and progression to NASH, and in studying potential anti-steatotic drugs. Additionally, the model could be used to investigate drug-induced hepatic steatosis, as both micro- and macrovesicular steatosis have been observed after in vitro and in vivo exposure to drugs such as glucocorticoids and antimicrobials [70].

## Figures and Tables

**Table 1:** Culture media.

Medium	Additives	Final Concentration of Additives			
		Glucose	Fructose	Insulin	Palmitic Acid
CTR	None	11 mM			
G	Glucose	25 mM			
F	Fructose		5 mM		
GF	Glucose and fructose	25 mM	5 mM		
GFI	Glucose, fructose, and insulin	25 mM	5 mM	1 nM	
GFIP	Glucose, fructose, insulin, and palmitic acid	25 mM	5 mM	1 nM	240 μM

CTR = control; G = glucose; F = fructose; GF = glucose and fructose; GFI = glucose, fructose and insulin; GFIP = glucose, fructose, insulin and palmitic acid.

## Supplementary Materials

The following are available online at https://www.mdpi.com/2072-6643/11/3/507/s1. Table S1: Primer-probes for lipid metabolism, Figure S1: Morphology of PCLSs incubated in medium without insulin, Figure S2: Ratio of fat droplets per nucleus in CTR PCLSs over time, Figure S3: Expression of *Acaca* and *Acacb* mRNA in CTR PCLSs over time, Figure S4: Expression of *Hspa5* mRNA in CTR PCLS over time.
