# Supplementary material for: A Pathophysiological Model of Non-Alcoholic Fatty Liver Disease Using Precision-Cut Liver Slices
Source: Nutrients. 2019 Feb 27;11(3):507. doi: 10.3390/nu11030507 (PMC6470479; doi:10.3390/nu11030507)
Supplement: Supplementary file 1 [file nutrients-11-00507-s001.pdf]

Supplementary data

Table S1. Taqman primer-probes and SYBR Green primers.

**Taqman primer-probes**				
**Abbreviation**	**Gene Symbol**	**Gene name**	**GenBank**	**UniGene ID**
Acaca	Acaca	Acetyl-CoA carboxylase alpha	EF121986.1	Rn.163753
Acacb	Acacb	Acetyl-CoA carboxylase beta	AB004329.1	Rn.162151
Mlxipl	Mlixpl	MLX interacting protein-like	AB074517.1	Rn.144656
Srebf1	Srebf1	Sterol regulatory element binding transcription factor 1	AF286469.2	Rn.198857
Srebf2	Srebf2	Sterol regulatory element binding transcription factor 2	BC101902.1	Rn.41063
Cpt1	Cpt1a	Carnitine palmitoyltransferase 1a, liver	BC072522.1	Rn.2856
Cpt2	Cpt2	Carnitine palmitoyltransferase 2	J05470.1	Rn.11389
Ywhaz	Ywhaz	Tyrosine 3-monooxygenase/tryptophan 5-monooxygenase activation protein, zeta	BC094305.1	Rn.1292
Il6	Il6	Interleukin 6	M26744.1	Rn.9873
Tgfb	Tgfb	Transforming growth factor beta	NM_021578.2	Rn.40136
Acta2	Acta2	Actin, alpha 2, smooth muscle, aorta	BC158550.1	Rn.195319
Col1a1	Col1a1	Collagen, type 1, alpha 1	BC133728.1	Rn.2953
		Size: FAM:S 250 rxn		
**SYBR Green primers**				
Abbreviation	Gene Symbol	Gene name	Forward	Reverse
Hspa5	Hspa5	Heat shock protein family A member 5	5′-CTGTGAGACACCTGACCGAC	5′-GACGCAGGAATAGGTGGTCC
Tnf	Tnf	Tumor necrosis factor-alpha	5′-ATGTGGAACTGGCAGAGGAG	5′-ACGAGCAGGAATGAGAAGAGG
Ywhaz	Ywhaz	Tyrosine 3-monooxygenase/tryptophan 5-monooxygenase activation protein, zeta	5′-TTGAGCAGAAGACGGAAGGT	5′-GAAGCATTGGGGATCAAGAA

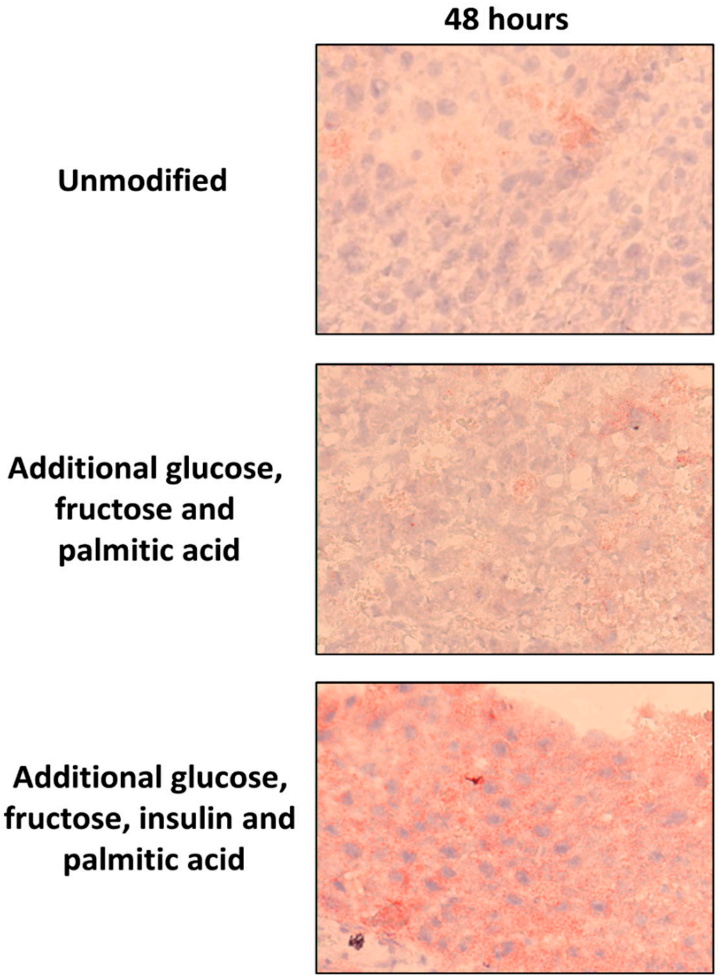

Figure S1. Morphology of PCLS incubated in medium without insulin. Representative Oil Red O stained sections of PCLS cultured in the modified culture medium for 48 hours (×400).

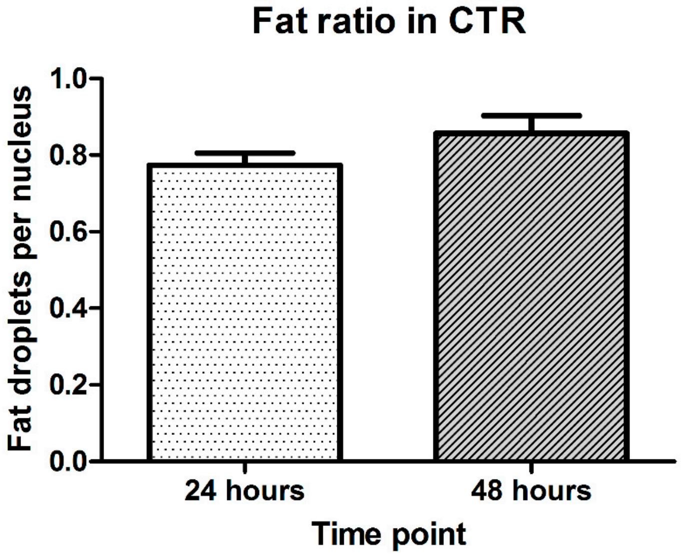

Figure S2. Ratio of fat droplets per nucleus in CTR PCLS over time. Data is expressed as mean ratio ±SEM (*N* = 4). A student's T-test comparing the two time points was used to determine significance.

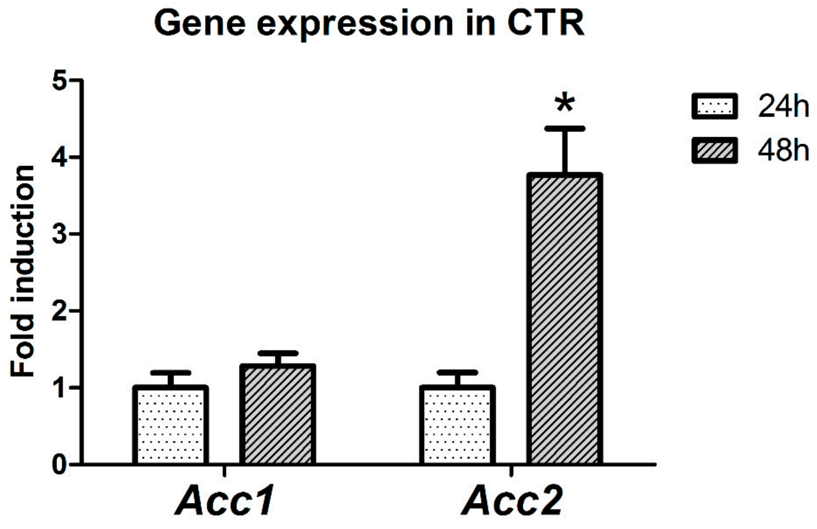

Figure S3. Expression of *Acaca* and *Acacb* mRNA in CTR PCLS over time. Data is expressed as mean relative fold induction ± SEM (N = 3). A student's T-test comparing fold inductions of the two time points was used to determine significance.

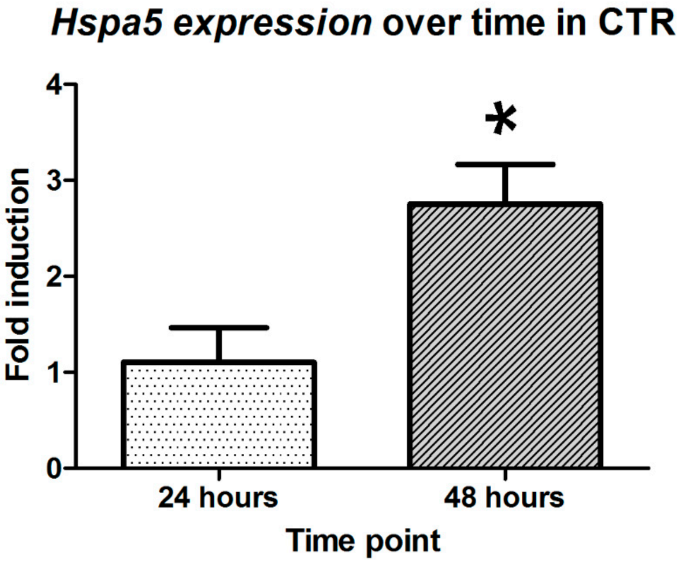

Figure S4. Expression of *Hspa5* mRNA in CTR PCLS over time. Data is expressed as mean relative fold induction ± SEM (N = 3). A student's T-test comparing fold inductions of the two time points was used to determine significance.
